# The Effect of Acrylic Surface Preparation on Bonding Denture Teeth to Cellulose Fiber-Reinforced Denture Base Acrylic †

**DOI:** 10.3390/jfb13040183

**Published:** 2022-10-10

**Authors:** Joanna Taczała-Warga, Jacek Sawicki, Michał Krasowski, Jerzy Sokołowski

**Affiliations:** 1 Institute of Materials Science and Engineering, Lodz University of Technology, Stefanowskiego 1/15, 90-924 Lodz, Poland; 2 University Laboratory of Material Research, Medical University of Lodz, Pomorska 251, 92-213 Lodz, Poland; 3 Department of General Dentistry, Medical University of Lodz, Pomorska 251, 92-213 Lodz, Poland

**Keywords:** PMMA, shear strength, tensile strength, mechanical surface development, dental techniques, acrylic resin

## Abstract

Patients who require dental prosthetic restoration using frame dentures in the front part of the mouth very frequently report that teeth fall out of their dentures. However, the available scientific papers are insufficient to compare the various methods of improving the connection between the denture base and the artificial tooth and choosing the best solution. This paper focuses on providing all parameters, enabling the reproduction of tests, and accounting for all variables. The paper uses an original method of creating grooves, sandpaper, sandblasting, and cutting the acrylate layer with a burr in one and two directions. Developed surfaces were additionally subjected to detailed examination. This study used 180 specimens divided into three groups and subjected to various environments (dry, artificial saliva, and thermocycles). Shearing and tensile strength tests were performed. The best results were obtained with a carbide burr. The increase in connection durability was as follows in the case of the shear test: 116.47% in dry samples, 155.38% in samples soaked in artificial saliva, and 46.59% in samples after thermocycles. The increase in tensile resistance was: 198.96% in a dry environment, 88.10% before being soaked in artificial saliva, and 94.04% after thermocycles.

## 1. Introduction

Dental acrylic resin is the primary material used in dental technology [1,2,3]. It is used to create removable braces, obturators, and complete or partial dentures. Acrylic resin includes mainly polymethyl methacrylate (PMMA) with additives that provide the material’s required hardness, color, or extended life [4].

Dentures consist of prefabricated artificial teeth and, in the case of complete dentures, a dental base plate. Where treatment requires a removable denture, there are additional metal components such as clasps and (in the case of removable denture metal frameworks) a palate arch or plate [5]. The presented research focused mainly on analyzing the connection between teeth and the dental base plate; therefore, only these elements will be further taken into account in this paper.

Three types of artificial teeth are available: ceramic, composite, and acrylic. Acrylic teeth are the most popular, as they are cheaper than the others and form the best connection with the acrylic base plate [4]. The dental base is also made of PMMA. Both materials differ in terms of other substances, such as coloring agents [6], but the primary material is the same. Artificial teeth are made of acrylic that has already been polymerized during the production stage. A dental technician creates an acrylic base plate by mixing liquid monomer and powder polymer.

The connection between the polymerized teeth and the polymerizing acrylic base plate is chemical [1,2,4,7,8]. Dental technicians use various procedures to improve it:

- Procedures enabling the creation of new chains and/or increasing the over-etching of the tooth’s structure (e.g., through using isobutyl methacrylate [9], dichloromethane [10], MMA [9,11], etc.), ready-made mixes sold as “glues” [9,12,13,14,15]), or rubbing the tooth structure with a selected chemical reagent;
- Mechanical modification of the surface, wherein technicians create retainers [16,17,18,19] or develop the surface [9,14,19,20,21,22,23,24];
- A combination of the two above methods (which is even suggested by manufacturers [15]).

However, multiple factors affect the quality of connections between the above elements. The above modifications of tooth surfaces give satisfying results provided that the following conditions are met [4,16,17]:

- The patient is missing two or more teeth;
- The force exerted on the tooth acts along the axis of the tooth (such forces are present in the case of premolars and molars);
- The surface of contact between the artificial tooth and the dental base is quite large (again, such surfaces are present in the case of premolars and molars);
- The teeth can be more deeply embedded into the dental plate (possibly in complete acrylic dentures).

In these conditions, the force is distributed across the tooth surface regularly enough to reduce the probability of a tooth being knocked out to a minimum. A problem appears when conditions significantly increasing the likelihood of a tooth coming loose are present. These include a thin layer of acrylic directly underneath the tooth, a small area of contact between the tooth and the dental plate, and the effects of forces acting at an angle to the tooth axis. These difficulties must be dealt with during the prosthetic restoration of individual missing teeth in the front part of the mouth using a denture framework [5].

Denture frameworks are much more expensive than complete acrylic dentures. Therefore, despite the higher price, patients receive a product that is much more susceptible to damage. The literature indicates that the problem is quite common; as many as 33% of denture repairs result from teeth being knocked out from dentures, with 29% of these incidents involving front teeth [20,25]. As a result, dentures that should serve their users for several years require repair after only a few months.

Another problem in this area is the difficulty in comparing experimental data. Numerous studies have analyzed the impact of acrylic tooth surface modifications on the quality of their connection with the acrylic denture base. Unfortunately, researchers do not use a single research model, and standards have been modified numerous times or do not reflect the actual situation and forces present in the mouth. The authors of the presented paper intended to organize the knowledge available in this area, enable a comparison of results, and specify the exact parameters of the best possible surface modification.

Therefore, this paper aims to analyze various mechanical surface development methods and their impact on the connection between polymerized acrylic and acrylic in the course of polymerization. The authors intended to determine the specific surface modification (its parameters, shape, etc.) that significantly impacts the strength of the connection between the materials. We decided to disregard the impact of chemical modification at this stage, as only a detailed analysis of individual modifications may answer the question of which method gives the best results.

The article also discussed the influence of the environment of the oral cavity on the quality of the connection.

## 2. Materials and Methods

Work began with creating forms using a 3D printer to prepare samples of suitable shapes for shear and tensile strength testing. The two tests were chosen to be incisors subjected to a shearing force, which knocks the teeth out, and a tensile force, which contributes to the tooth becoming unstuck from the base [4,17]. Samples and tests followed the standard application of dental materials used in dental prostheses (ISO 22112:2017). The material imitating artificial teeth was made of commercial Vertex Rapid Simplified heat-cured acrylic resin (Vertex Dental, Soesterberg, The Netherlands). The material imitating the dental base was an original acrylic composite reinforced with cellulose fibers, described in detail in the article [26]. Various fillers may be used to reinforce PMMA, primarily made up of dental acrylic resin [27,28,29,30,31]. However, due to its lack of impact on the color of prostheses, biocompatibility, and the low market price of cellulose compared to other fillers, our original composite best meets the requirements expected from materials used in dental plates. In addition, the material is more durable than standard acrylic [32] and was devised as an element of a larger research project (of which this paper is a part).

**Figure 1:**
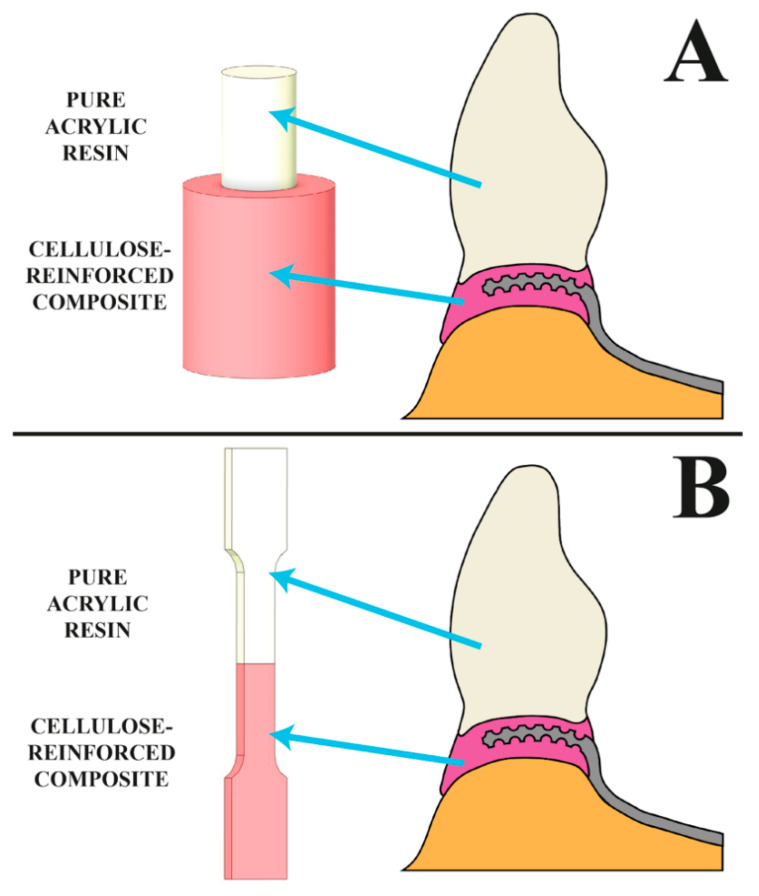
The visualization shows the samples’ shapes and corresponding components of a dental framework. Samples used in (**A**) shear testing, (**B**) tensile testing.

**Figure 2:**
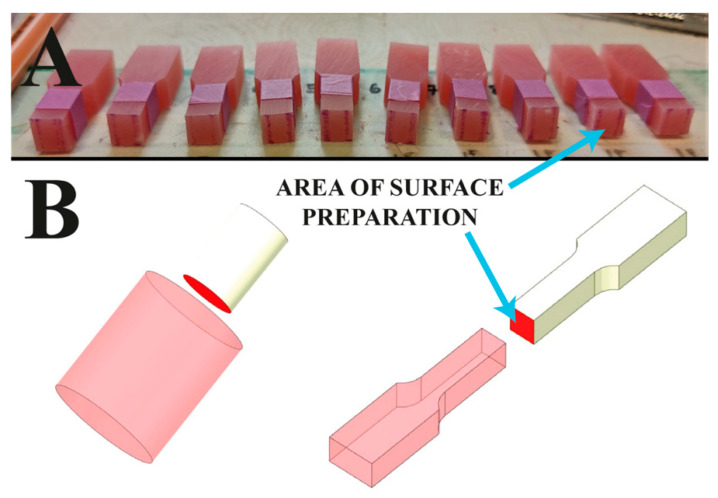
Location subjected to surface development shown on: (**A**) a photograph showing the prepared halves imitating a tooth, (**B**) a visualization showing the connection with the component imitating a dental base (figure from in the Ph.D. thesis: The influence of cellulose on the properties of methacrylic polymers for dental applications, J. Taczała-Warga).

The test samples were made up of two parts (Figure 1). The part imitating an acrylic tooth was the first to be created (Figure 2). The next stage was the modification of its surface using selected methods of mechanical surface development. As a result, the sample was consistent with a tooth in the preparation stage before the polymerization process. All prepared surfaces were also subjected to microgeometry testing with the contact profilometry method using a type S neox 3D Sensofar optical profilometer manufactured by Terrassa (Barcelona, Spain) and energy-dispersive X-ray spectroscopy (EDS) using a JSM-6610LV Scanning Electron Microscope (SEM) manufactured by JEOL (Peabody, MA, USA). Before preparing the second part of the sample, all surfaces were cleaned with a jet of warm water and dried (to prevent any dirt on the surface). The samples were not treated with any other chemical reagents. The samples prepared this way were again placed in the previously printed molds and finished using acrylic composite to obtain a complete sample.

All samples were created and processed by a single person to avoid the impact of human error on the tests.

The research included all examined surface modifications based on surface development so far:

- Cutting a layer of glazing using a burr [9,20,21,22,23] (group CB_III and CB_#);
- Sandblasting [9,11,14,19,20,21,22,23,24,33,34,35,36] (group SB);
- Using sandpaper [37,38,39,40] (group SP).

Retainers were not included in the study as their size prevents an objective comparison with the surface development method. Furthermore, they have already been analyzed by authors of papers [16,17] and do not affect connection quality.

The above surface development methods were compared with our original modification method—grooving (group G)—and control samples without surface modification. Rifling parameters were chosen based on experimentally tested numerical simulations described in detail in the literature [41]. A prosthetic diamond disc with a thickness of 0.20 mm was used to create the grooves. Finally, two grooves were created at a distance of 5.00 mm.

Table 1 shows all parameters of surface modification. The parameters used were set based on the above references.

All samples were additionally divided into three groups based on the impact of the oral environment:

- No impact (group D);
- After thermocycles [14,21,42,43,44,45] (group T);
- After soaking in artificial saliva [9,21,44,45,46,47] (group AF).

A total of 180 samples were prepared. The entire division and marking of samples are shown in Table 2.

Thermocycles were carried out using a Thermocykler THE1100e device, manufactured by SD Mechatronik GmbH (Feldkirchen-Westerham, Germany), using the following settings: 5000 cycles, a temperature between 5 °C and 55 °C, drying time 10 s. The settings used were chosen and averaged based on research covering similar issues [14,21,42,43,44,45].

Artificial saliva was prepared based on a mixture described by Fusayama–Meyer [48,49,50,51,52]. Its composition and proportions are shown in Table 3. The ingredients were diluted in distilled water. All reagents were manufactured by Chempur (Piekary Śląskie, Poland). The samples created were soaked for 48 h at 37 °C before testing [9,38,47].

**Figure 3:**
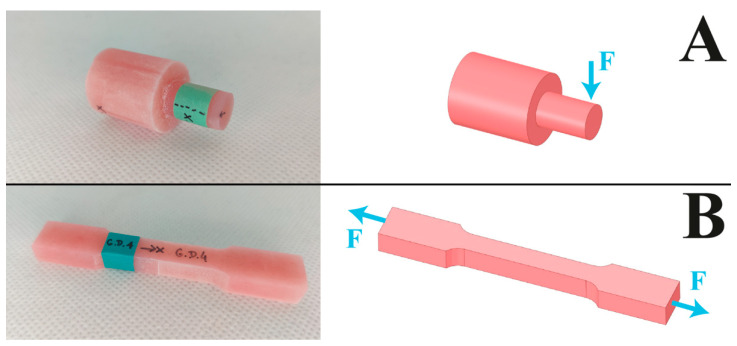
Diagram of the direction of force during strength testing: (**A**) shear test, (**B**) tensile test. Photographs on the left show the appearance of the final samples.

Strength (shear and tensile) tests were performed using a universal testing machine (Zwick/Roell) following standards used in dentistry and dental technology (ISO 22112:2017). The machine knife displacement rate was set to 2 mm/min. The results were statistically analyzed using Origin 2020 statistical software (OriginLab Corporation, Northampton, MA, USA). The individual research hypotheses were tested using the 2-way ANOVA test, with statistical significance assumed at a level of *p* = 0.05, and the HSD Tukey’s post hoc test was applied in statistical analyses. The direction of the force is shown in Figure 3.

Following the strength tests, fissures in all samples were analyzed using a VHX confocal microscope manufactured by Keyence International (Mechelen, Belgium).

## 3. Results

### 3.1. 3D Microscope Observations by Profilometer

**Figure 4:**
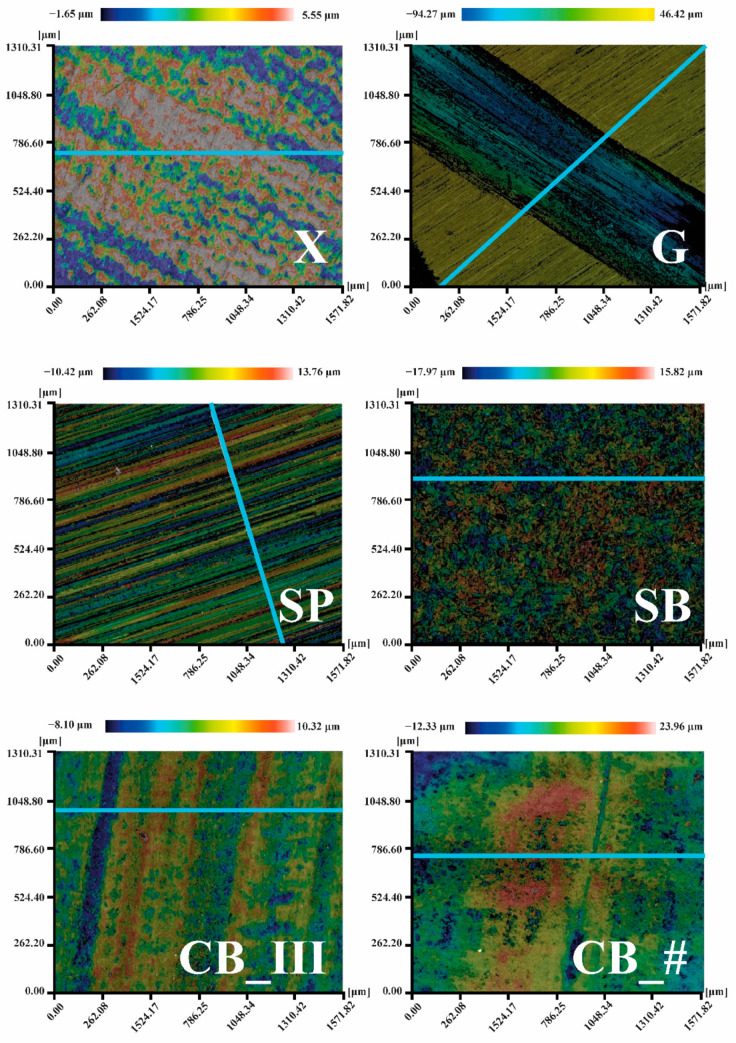
Deformation maps for all surface preparation methods were obtained using a 3D profilometer. The blue line marks the sections used to measure the Ra parameter (figure from the Ph.D. thesis: The influence of cellulose on the properties of methacrylic polymers for dental applications, J. Taczała-Warga).

**Figure 5:**
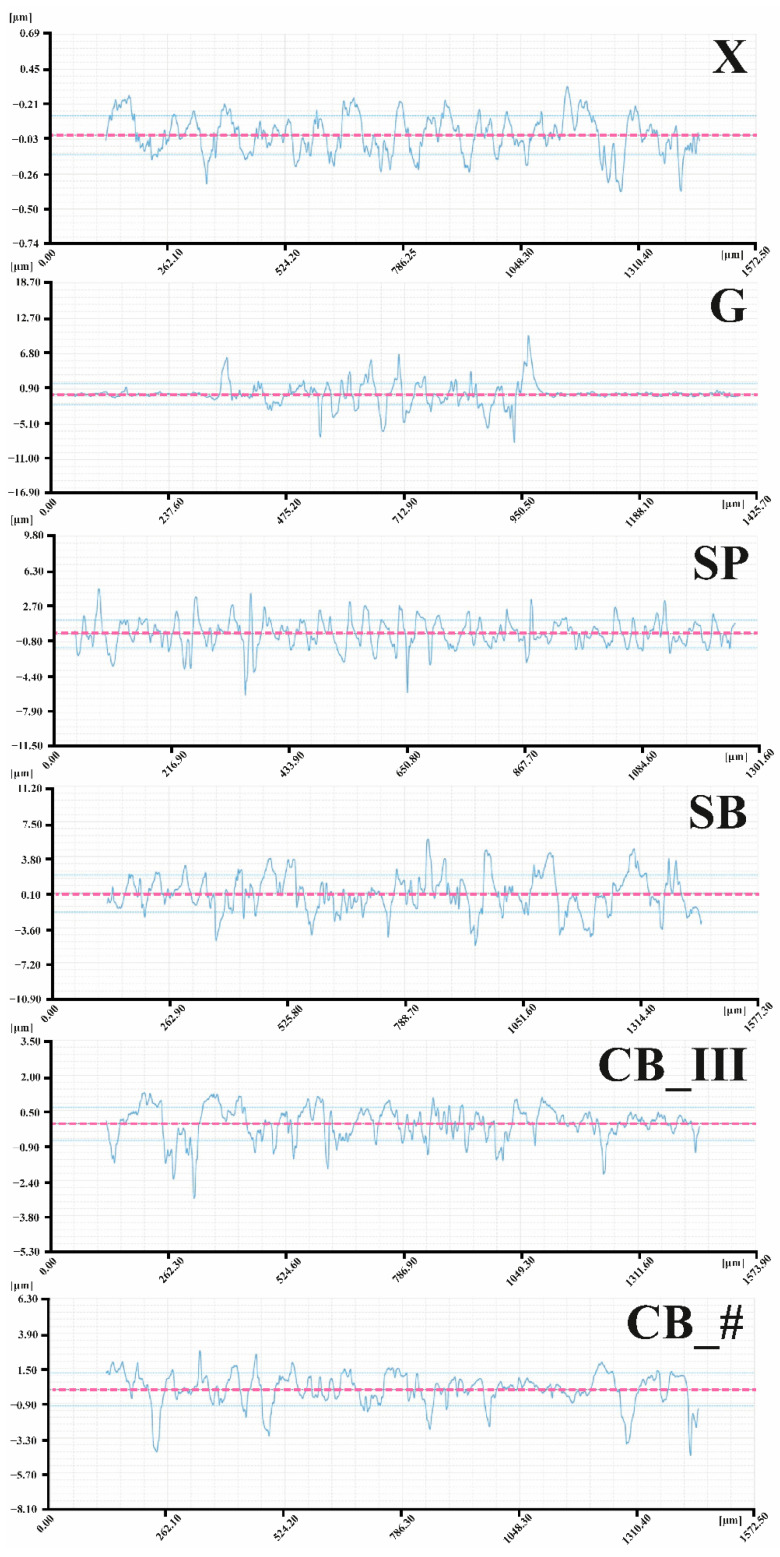
Chart of Ra roughness measured along the blue lines marked in Figure 4 (figure from the Ph.D. thesis: The influence of cellulose on the properties of methacrylic polymers for dental applications, J. Taczała-Warga).

All measurements were made on the same surface area (1.57 × 1.32 mm). The test produced colorful maps of sample surfaces (Figure 4) and 2D charts of the average arithmetic deviation of the roughness profile (i.e., the Ra parameter (Figure 5)), calculated following standard ISO 4287. Ra values were compared to measured values of the following 3D parameters: average arithmetic deviation of the height of surface unevenness from the reference plane (Sa) and average square deviation of the surface unevenness from the reference plane (Sq), determined following standard ISO 25178. The obtained values enabled a detailed assessment of the micro geometry created on the surface of the samples [53,54].

Sa and Sq values for all tested groups was as follows:

- X—Sa 0.26 μm, Sq 0.33 μm;
- G—Sa 42.84 μm, Sq 45.39 μm;
- SP—Sa 2.17 μm, Sq 2.72 μm;
- SB—Sa 2.86 μm, Sq 3.60 μm;
- CB_III—Sa 0.79 μm, Sq 1.04 μm;
- CB_#—Sa 1.54 μm, Sq 1.99 μm.

**Figure 6:**
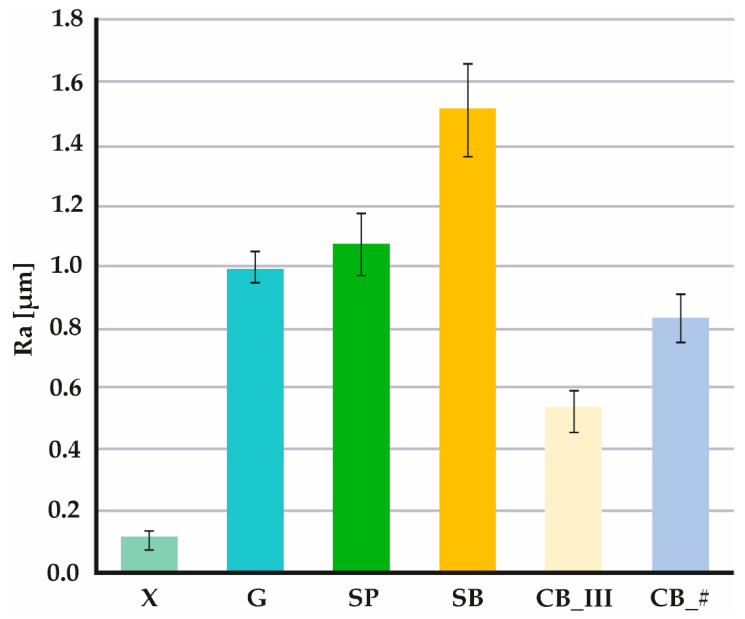
Values of the Ra parameter for each surface development method (figure from the Ph.D. thesis: The influence of cellulose on the properties of methacrylic polymers for dental applications, J. Taczała-Warga).

The results of the Ra parameter are shown in Figure 6. The following average values were recorded: for X group 0.11 μm, for G group 0.99 μm, for SP 1.51 μm, for CB_III 0.53 μm, and for CB_# 0.83 μm.

### 3.2. EDS

**Figure 7:**
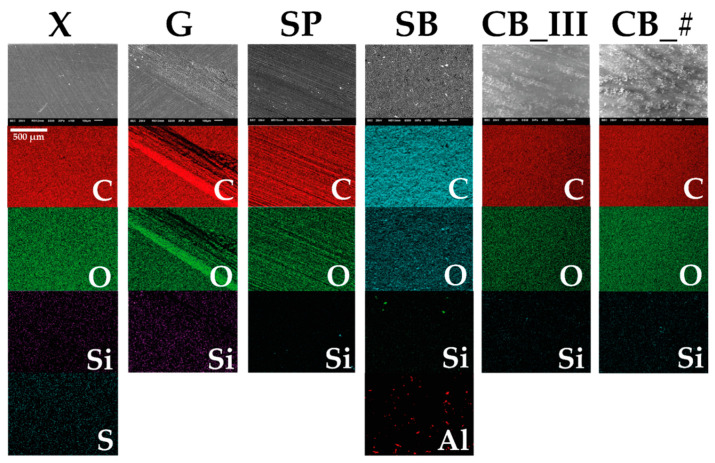
EDS analyses obtained and corresponding SEM images (figure from the Ph.D. thesis: The influence of cellulose on the properties of methacrylic polymers for dental applications, J. Taczała-Warga).

**Figure 8:**
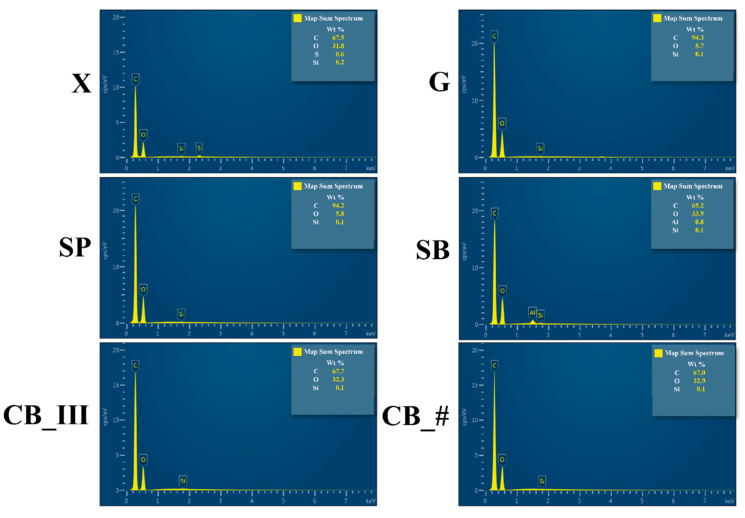
Measurements of spectrums of each element from the EDS analysis (figure from the Ph.D. thesis: The influence of cellulose on the properties of methacrylic polymers for dental applications, J. Taczała-Warga).

Examination under SEM and EDS analyses (Figure 7 and Figure 8) were carried out to characterize all mechanical preparations of sample surfaces better. Measurements were performed at the following device settings: 20 kV, 30 Pa, magnification ×100 in the case of SEM, and ×500 in the case of EDS.

### 3.3. Strength Tests

**Figure 9:**
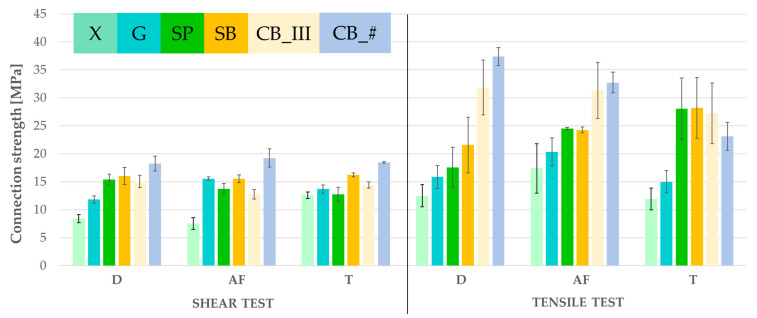
Shear and tensile test results (figure from the Ph.D. thesis: The influence of cellulose on the properties of methacrylic polymers for dental applications, J. Taczała-Warga).

Two strength tests were performed to obtain results mirroring, as closely as possible, the natural conditions in the mouth and outside. The samples were subjected to shearing and tensile force. The results are presented in graphical form in Figure 9.

Detailed results of both tests are also presented in tabular format (Table 4).

The performed statistical analysis of the variance of the results (2-way ANOVA) of the shear and tensile strength tests showed statistically significant differences between the mean (i.e., mean shear and tensile strengths) in the groups of samples prepared with different methods (*p* < 0.05). At the same time, the results of the F test with the adopted level of significance (*p* = 0.05) indicate no grounds to reject the hypothesis that there are no differences between averages (i.e., average shear and tensile strengths) in the groups subjected to conditions in different environments. This means that the type of environment (AF—saliva, T—thermal shocks) has no significant effect on the properties of the tested elements. The analysis of variance with the adopted significance level (*p* = 0.05) also did not reveal any interaction between the type of sample preparation (method) and the type of environment in which it was then present. Tukey’s post-hoc test of the shear strength test results (Table A1) showed statistically significant differences between the means subjected to different treatments (groups G, SP, SB, CB_III, CB_ #) and the reference group (group X—samples untreated). This means that each type of treatment tested significantly improved the tensile strength of the samples compared to the reference group. In the case of tensile strength (Table A2), the most significant differences were observed for the treated CB_ # vs. the untreated samples (group X).

### 3.4. Observation of Fissures

**Figure 10:**
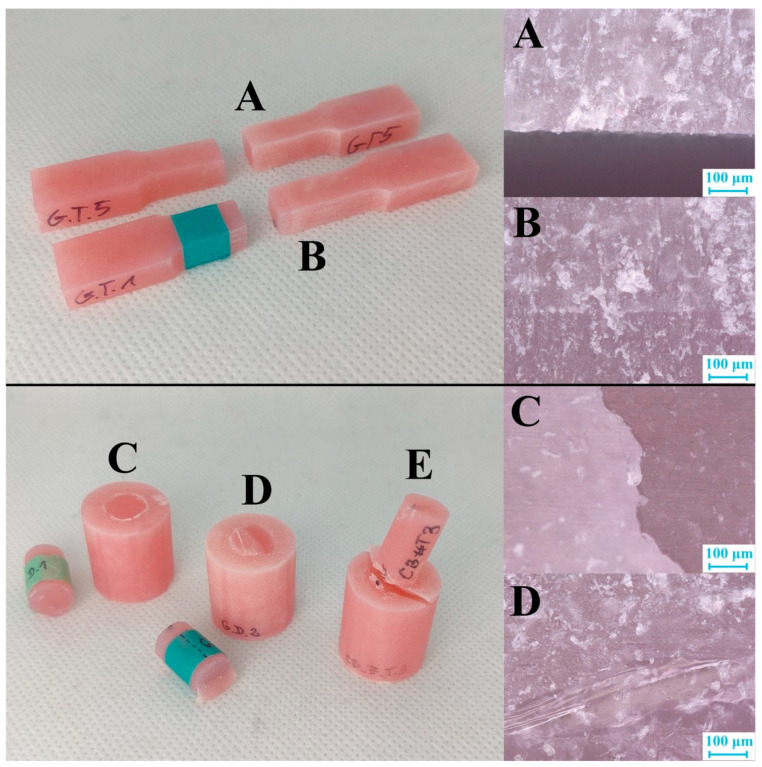
Representations of all types of cracks observed during the test. Photos of the samples are on the left, and photos of fissures made using a confocal microscope are on the right. Tensile test: (**A**) crack at the connection; (**B**) crack deep inside the material. Shear test: (**C**) crack at the connection in X group samples; (**D**) crack deep in the material; (**E**) no complete breakage (figure from the Ph.D. thesis: The influence of cellulose on the properties of methacrylic polymers for dental applications, J. Taczała-Warga).

All types of damage observed with the microscope are shown in Figure 10.

## 4. Discussion

By analyzing the obtained data, we can confirm that a smooth sample surface subjected to no mechanical processing (control sample marked as X) has the lowest Ra roughness coefficient. The tested value of this parameter in the sample was 0.11 μm. The same sample also had the lowest Sa (0.26 μm) and Sq (0.33 μm) parameters. The literature data also confirm that the test was performed correctly, as the measured Ra roughness parameter for an entire acrylic surface was 0.12 μm [55].

The average Ra coefficient for a grooved sample (G) is 0.99 μm. However, given the information shown in Figure 4 and Figure 5 and the results of the Sa (42.84 μm) and Sq (45.39 μm) measurements, it is apparent that the roughness value in the groove itself is much higher than on an untreated surface. Roughness inside the groove ranges from approximately 8.00 μm to approx. 9.00 μm on the chart, with the entire groove forming a straightforward step. This range of values was measured at a depth below the untreated surface of the sample.

The parameters measured on the surface of the sample treated with sandpaper (SP) of Ra 1.07 μm were also nearly identical to results found in the literature, with the Ra coefficient measured at 1.03 μm [56].

The highest Ra coefficient was recorded in sandblasted samples (SB) = 1.51 μm, Sa 2.86 μm, and Sq 3.60 μm. Up to this point, an upwards trend of the Ra coefficient had been recorded. Samples prepared using a prosthetic carbide burr had lower Ra coefficients. Cutting the resin layer in one direction (CB_III) resulted in a Ra value of 0.53 μm, and in two directions (CB_#) produced a Ra value of 0.83 μm. Of note, however, is that in the case of these two methods, peaks with extreme negative values were predominant (positive values are not that extreme). The results differ from those presented by other researchers (Ra 1.52 μm in reference [56]). However, the materials and techniques used play a vital role when comparing sanding and burring. Many papers do not provide information on the angle used when sandblasting, while research shows that this aspect is essential in the context of connecting materials [34,35]. The situation is similar concerning the choice of burrs. In their articles, researchers only specify that the external layer was cut using a cemented carbide burr. However, burrs are available in numerous shapes, with slots made using various methods and in various thicknesses, and may also be made of multiple alloys or coated with additional material [57]. In our paper, we provide all parameters to enable researchers to reproduce and compare the tests and to allow dental technicians to choose precisely the same method as the one used by us to achieve their intended goal, namely improving the quality of connection. As a result, parameters provided by other researchers are insufficient to compare results reliably.

An analysis of surfaces prepared using the EDS method confirmed that the main elements, carbon (C) and oxygen (O), were abundant in all samples. These are the essential elements found in polymethyl methacrylate. In addition, silicon (Si) was found in all groups. Silicon is one of the elements often found among ingredients of commercial acrylic resin [58]. Silica is used as a filler.

Other elements detected in various samples include sulfur (S) in the control sample marked as X and aluminum (Al) in sample SB. As S is another element present in fillers [59], we can assume that a significant amount of sulfur is deposited outside the material during polymerization and mechanical treatment causes this layer to be destroyed. Another possibility is that S is present in all samples, but only in the case of the X group sample was the amount of sulfur in the examined area sufficient to be detected. Al was another identified element. As Al was found in the sandblasted sample marked as SB, and Al_2_O_3_ was the material used for sandblasting, we can conclude that this treatment method causes sand particles to become embedded in the material. This may create something like hooks filled with acrylic from the denture base (in this case, composite from the second part of the sample).

As the SP sample did not contain a more significant amount of Si than the other samples, we can assume that fragments of sandpaper did not become embedded during treatment, as the material used in the testing was made of SiC.

As predicted, both tests of the X control samples yielded the lowest results. This shows how important it is to correctly prepare the tooth’s surface before connecting it with acrylic. Regarding the impact of the environment, artificial saliva reduced the shear strength of the X samples but increased their tensile strength. The opposite was observed about the effect of thermocycles.

Considering all of the results, the following upward trend was observed: G, SP, SB, CB_III, CB_#. The highest values in tensile and shear tests were measured in samples treated using a cemented carbide burr. The highest strength jump occurs in tensile tests after subjecting the samples to thermocycles. To avoid this jump, the patient can be told to avoid eating very hot/cold food or to take extended breaks between consuming these foods or beverages. The percentage increase of durability of connections made using this method compared to control samples is as follows in the case of shear resistance: increase by 116.47% in dry samples, 155.38% in samples soaked in artificial saliva, and 46.59% in samples subjected to thermocycles. The increase of tensile resistance in the samples was as follows: 198.96% in dry samples, 88.10% in samples soaked in artificial saliva, and 94.04% in samples subjected to thermocycles.

It would seem that the high roughness coefficient of the SB samples should positively impact the quality of the connection. However, the results of the test indicate otherwise. There may be two reasons for this. The first is that excessive differences in surface heights may increase the material’s susceptibility to cracking, and the second is the presence of aluminum oxide particles embedded in the sample’s surface. The hooks created this way do not improve the adhesion of the acrylic and may cause air bubbles to appear. This, as well as the presence of a material other than acrylic, may reduce the area of connection between the acrylic and the second part of the sample.

The last stage of the test was the observation of fissures under a microscope. The examination did not indicate any significant impact of the surface preparation on the manner of cracking. The only correlation was the smooth cracks at the connection of both materials in all X samples. Mechanical modification of the samples caused cracks to appear deep inside the pure acrylic or acrylic composite.

The presence of cracks deep in the material means that the durability of the connection was more significant than the material’s durability. Furthermore, it is worth noting that no samples were destroyed (apart from samples from the X group). This means that any mechanical modification increases the strength of the connection between the materials and may contribute to the gradual propagation of cracks [41]. The appearance of a crack will, therefore, not prevent the patient from continuing to use the denture until a tooth is entirely knocked out.

## 5. Conclusions

In conclusion, we may clearly state that any mechanical modification significantly impacts the connection between a dental base and an artificial tooth.

To further improve the quality of connection where increased durability is required, dental technicians should prepare dentures using a cemented carbide burr. This method will significantly improve the quality of the connection. To further extend the life of such a connection, the patient should avoid eating very hot/cold food or take long breaks between consuming these foods or liquids.

We note that in the study presented above, we focused solely on mechanical methods of surface development to ensure that our research of this method is as thorough as possible and other available research can be systematized. Future researchers and dental technicians now possess precise data enabling them to compare methods and use them in their laboratories.

Combining this technique with chemical surface modification may allow us to obtain even better properties and will be the subject of our future research.

## Figures and Tables

**Table 1:** Applied surface modifications and their parameters.

Preparation of Surface	Symbol of the Group	Parameters
Without modification	X	-
Grooving	G	Width 0.20 mm, thickness 0.10 mm, the distance between the grooves 5.00 mm, force placed at an angle of 90°, speed 30,000 RPM.
Sandpaper	SP	Surface roughness by SiC (silicon carbide/carborundum) sandpaper with the gradation of 180 µm.
Sandblasting	SB	Sandblasting by Al_2_O_3_ with 110 µm particle size, at the 45°, the distance from the nozzle 15 mm and 0.4 MPa.
Carbide bur-parallel	CB_III	Carbide bur used in dental techniques with the same shape on whole geometry, red color, no. F93, L 16.0 mm, ⌀ 023 mm. Surface roughened in one direction.
Carbide bur-cross	CB_#	Carbide bur used in dental techniques with the same shape on whole geometry, red color, no. F93, L 16.0mm, ⌀ 023mm. Surface roughened in two directions.

**Table 2:** Legend for marking and dividing samples.

Preparation of Surface	Environmental Impact		
	D—Dry (No Environmental Impact)	AF—Artificial Saliva	T—Thermocycling
X—control specimen, no modifications	X.D, 5 x shear test X.D, 5 x tensile test	X.AF, 5 x shear test X.AF, 5 x tensile test	X.T, 5 x shear test X.T, 5 x tensile test
G—grooves	G.D, 5 x shear test G.D, 5 x tensile test	G.AF, 5 x shear test G.AF, 5 x tensile test	G.T, 5 x shear test G.T, 5 x tensile test
SP—sandpaper	SP.D, 5 x shear test SP.D, 5 x tensile test	SP.AF, 5 x shear test SP.AF, 5 x tensile test	SP.T, 5 x shear test SP.T, 5 x tensile test
SB—sandblasting	SB.D, 5 x shear test SB.D, 5 x tensile test	SB.AF, 5 x shear test SB.AF, 5 x tensile test	SB.T, 5 x shear test SB.T, 5 x tensile test
CB_III—preparation with carbide bur in one direction	CB_III.D, 5 x shear test CB_III.D, 5 x tensile test	CB_III.AF, 5 x shear test CB_III.AF, 5 x tensile test	CB_III.T, 5 x shear test CB_III.T, 5 x tensile test
CB_#—preparation with carbide bur in two directions	CB_#.D, 5 x shear test CB_#.D, 5 x tensile test	CB_#.AF, 5 x shear test CB_#.AF, 5 x tensile test	CB_III.T, 5 x shear test CB_III.T, 5 x tensile test

**Table 3:** Composition of the artificial saliva.

Composition	CAS No	Concentration (g/L)
Potassium chloride (KCl)	7447-40-7	0.400
Sodium chloride (NaCl)	7647-14-5	0.400
Calcium chloride dihydrate (CaCl_2_·2H_2_O)	10035-04-8	0.906
Monosodium phosphate dihydrate (NaH_2_PO_4_·2H_2_O)	13472-35-0	0.690
Sodium sulfide nonahydrate (Na_2_S·9H_2_O)	1313-84-4	0.005
Urea (CH_2_N_2_O)	57-13-6	1.0

**Table 4:** Results of the shear test of the connection.

Preparation of Surface	Shear Test [MPa]						Tensile Test [MPa]					
	D		AF		T		D		AF		T	
	a.s.	s.d.	a.s.	s.d.	a.s.	s.d.	a.s.	s.d.	a.s.	s.d.	a.s.	s.d.
X	8.70	0.67	7.74	0.56	12.60	0.15	12.64	1.51	18.48	0.20	12.24	0.60
G	11.60	0.73	15.86	1.02	13.78	0.59	15.76	2.00	21.40	4.41	18.24	1.95
SP	14.85	1.33	12.45	1.64	12.70	0.12	18.77	1.59	24.50	1.84	26.36	2.49
SB	15.54	0.10	15.72	1.40	16.12	1.30	21.26	0.56	23.16	3.85	28.44	5.45
CB_III	15.08	0.55	13.78	0.09	14.96	0.05	32.94	5.39	29.40	2.87	26.38	6.75
CB_#	17.78	0.67	19.54	0.56	18.58	0.15	37.62	1.51	31.30	0.20	24.46	0.60

a.s.—average strength; s.d.—standard deviation.

## Appendix A

Results of performed HSD Tukey’s statistical tests are shown below, with the shear test in Table A1 and the tensile test in Table A2.

**Table A1:** HSD Tukey’s test; variable shear stress. Approximate probabilities for post hoc tests.

	Surface Preparation	Environment	1	2	3	4	5	6	7	8	9	10	11	12	13	14	15	16	17	18
1	X	D		1.00	0.64	0.95	0.01	0.20	0.04	0.71	0.60	0.01	0.01	0.00	0.03	0.20	0.03	0.00	0.00	0.00
2	X	AF	1.00		0.26	0.66	0.00	0.05	0.01	0.31	0.23	0.00	0.00	0.00	0.00	0.05	0.01	0.00	0.00	0.00
3	X	T	0.64	0.26		1.00	0.87	1.00	1.00	1.00	1.00	0.94	0.91	0.79	0.99	1.00	0.99	0.17	0.01	0.05
4	G	D	0.95	0.66	1.00		0.49	1.00	0.88	1.00	1.00	0.63	0.55	0.38	0.81	1.00	0.85	0.04	0.00	0.01
5	G	AF	0.01	0.00	0.87	0.49		1.00	1.00	0.83	0.90	1.00	1.00	1.00	1.00	1.00	1.00	1.00	0.73	0.97
6	G	T	0.20	0.05	1.00	1.00	1.00		1.00	1.00	1.00	1.00	1.00	0.99	1.00	1.00	1.00	0.60	0.07	0.28
7	SP	D	0.04	0.01	1.00	0.88	1.00	1.00		0.99	1.00	1.00	1.00	1.00	1.00	1.00	1.00	0.94	0.32	0.71
8	SP	AF	0.71	0.31	1.00	1.00	0.83	1.00	0.99		1.00	0.91	0.87	0.74	0.98	1.00	0.99	0.14	0.01	0.04
9	SP	T	0.60	0.23	1.00	1.00	0.90	1.00	1.00	1.00		0.96	0.93	0.83	0.99	1.00	1.00	0.20	0.01	0.06
10	SB	D	0.01	0.00	0.94	0.63	1.00	1.00	1.00	0.91	0.96		1.00	1.00	1.00	1.00	1.00	1.00	0.60	0.93
11	SB	AF	0.01	0.00	0.91	0.55	1.00	1.00	1.00	0.87	0.93	1.00		1.00	1.00	1.00	1.00	1.00	0.68	0.96
12	SB	T	0.00	0.00	0.79	0.38	1.00	0.99	1.00	0.74	0.83	1.00	1.00		1.00	0.99	1.00	1.00	0.83	0.99
13	CB_III	D	0.03	0.00	0.99	0.81	1.00	1.00	1.00	0.98	0.99	1.00	1.00	1.00		1.00	1.00	0.97	0.41	0.80
14	CB_III	AF	0.20	0.05	1.00	1.00	1.00	1.00	1.00	1.00	1.00	1.00	1.00	0.99	1.00		1.00	0.60	0.07	0.28
15	CB_III	T	0.03	0.01	0.99	0.85	1.00	1.00	1.00	0.99	1.00	1.00	1.00	1.00	1.00	1.00		0.96	0.36	0.76
16	CB_#	D	0.00	0.00	0.17	0.04	1.00	0.60	0.94	0.14	0.20	1.00	1.00	1.00	0.97	0.60	0.96		1.00	1.00
17	CB_#	AF	0.00	0.00	0.01	0.00	0.73	0.07	0.32	0.01	0.01	0.60	0.68	0.83	0.41	0.07	0.36	1.00		1.00
18	CB_#	T	0.00	0.00	0.05	0.01	0.97	0.28	0.71	0.04	0.06	0.93	0.96	0.99	0.80	0.28	0.76	1.00	1.00	

**Table A2:** HSD Tukey’s test; variable tensile stress. Approximate probabilities for post hoc tests.

	Surface Preparation	Environment	1	2	3	4	5	6	7	8	9	10	11	12	13	14	15	16	17	18
1	X	D		1.00	1.00	1.00	0.95	1.00	1.00	0.62	0.37	0.95	0.80	0.16	0.01	0.10	0.36	0.00	0.04	0.63
2	X	AF	1.00		1.00	1.00	1.00	1.00	1.00	1.00	0.98	1.00	1.00	0.86	0.28	0.75	0.98	0.03	0.49	1.00
3	X	T	1.00	1.00		1.00	0.92	1.00	1.00	0.57	0.32	0.93	0.75	0.13	0.01	0.08	0.31	0.00	0.03	0.57
4	G	D	1.00	1.00	1.00		1.00	1.00	1.00	0.95	0.79	1.00	0.99	0.51	0.08	0.38	0.79	0.00	0.18	0.95
5	G	AF	0.95	1.00	0.92	1.00		1.00	1.00	1.00	1.00	1.00	1.00	0.99	0.67	0.98	1.00	0.13	0.87	1.00
6	G	T	1.00	1.00	1.00	1.00	1.00		1.00	1.00	0.97	1.00	1.00	0.84	0.25	0.72	0.97	0.02	0.45	1.00
7	SP	D	1.00	1.00	1.00	1.00	1.00	1.00		1.00	0.99	1.00	1.00	0.89	0.31	0.79	0.99	0.03	0.53	1.00
8	SP	AF	0.62	1.00	0.57	0.95	1.00	1.00	1.00		1.00	1.00	1.00	1.00	0.96	1.00	1.00	0.44	1.00	1.00
9	SP	T	0.37	0.98	0.32	0.79	1.00	0.97	0.99	1.00		1.00	1.00	1.00	1.00	1.00	1.00	0.71	1.00	1.00
10	SB	D	0.95	1.00	0.93	1.00	1.00	1.00	1.00	1.00	1.00		1.00	0.99	0.65	0.97	1.00	0.12	0.85	1.00
11	SB	AF	0.80	1.00	0.75	0.99	1.00	1.00	1.00	1.00	1.00	1.00		1.00	0.88	1.00	1.00	0.28	0.97	1.00
12	SB	T	0.16	0.86	0.13	0.51	0.99	0.84	0.89	1.00	1.00	0.99	1.00		1.00	1.00	1.00	0.92	1.00	1.00
13	CB_III	D	0.01	0.28	0.01	0.08	0.67	0.25	0.31	0.96	1.00	0.65	0.88	1.00		1.00	1.00	1.00	1.00	0.96
14	CB_III	AF	0.10	0.75	0.08	0.38	0.98	0.72	0.79	1.00	1.00	0.97	1.00	1.00	1.00		1.00	0.97	1.00	1.00
15	CB_III	T	0.36	0.98	0.31	0.79	1.00	0.97	0.99	1.00	1.00	1.00	1.00	1.00	1.00	1.00		0.71	1.00	1.00
16	CB_#	D	0.00	0.03	0.00	0.00	0.13	0.02	0.03	0.44	0.71	0.12	0.28	0.92	1.00	0.97	0.71		1.00	0.44
17	CB_#	AF	0.04	0.49	0.03	0.18	0.87	0.45	0.53	1.00	1.00	0.85	0.97	1.00	1.00	1.00	1.00	1.00		1.00
18	CB_#	T	0.63	1.00	0.57	0.95	1.00	1.00	1.00	1.00	1.00	1.00	1.00	1.00	0.96	1.00	1.00	0.44	1.00	
